# Regulatory effects of *Auricularia cornea* var. Li. polysaccharides on immune system and gut microbiota in cyclophosphamide-induced mice

**DOI:** 10.3389/fmicb.2022.1056410

**Published:** 2022-11-02

**Authors:** Ming Zhao, Wei Shi, Xijun Chen, Yanfang Liu, Yan Yang, Xianghui Kong

**Affiliations:** ^1^Institute of Microbiology, Heilongjiang Academy of Sciences, Harbin, China; ^2^Key Laboratory of Flexible Electronics, Institute of Advanced Materials, Nanjing Tech University, Nanjing, China; ^3^China Technology Optimization (Heilongjiang) Technology Industry Co., Ltd., Harbin, China; ^4^Institute of Edible Fungi, Shanghai Academy of Agricultural Sciences, Shanghai, China

**Keywords:** *Auricularia cornea* var. Li., polysaccharides, immune system, gut microbiota, short-chain fatty acids (SCFAs)

## Abstract

The immuno-regulating potential of edible fungus polysaccharides has gained more and more attention. However, there is little information about the study of *Auricularia cornea* var. Li. polysaccharides regulating immunomodulatory activity. The objective of this work to analyze the immunomodulatory activity and the mechanism of *A. cornea* var. Li. polysaccharides supplementation in an immunosuppressed mice model induced by cyclophosphamide. The effects of *A. cornea* var. Li. polysaccharides on immune system including immune organ indices, immunoglobulin contents, and inflammation cytokines in immunosuppressed mice were determined. In addition, the regulatory effects of *A. cornea* var. Li. polysaccharides on the gut microbiota and their metabolites were analyzed. Results showed that *A. cornea* var. Li. polysaccharides significantly elevated immune organ indexes, remarkably enhanced the levels of immunoglobulin A (IgA), IgG and IgM in serum and secretory IgA (sIgA) in the intestinal mucosa, conspicuously stimulated the levels of tumor necrosis factor-α (TNF-α), interleukin-2 (IL-2), IL-4, and IL-10 in the serum. *A. cornea* var. Li. polysaccharides also could restore gut microbiota to the pattern that is similar with that of the control group with increase of the relative abundances of short-chain fatty acids (SCFAs)-producing bacteria. Furthermore, the content of SCFAs were increased after *A. cornea* var. Li. polysaccharides supplementation. This study provides useful information for applications of *A. cornea* var. Li. polysaccharides in immune-regulated foods and medicine.

## Introduction

The sub-health condition is a critical condition between health and illness. The causes are varied, including staying up late, living an irregular life, smoking, drinking, and other unhealthy lifestyles, which are all important in the development of the sub-health condition. Eventually, it can lead to a decrease in immune function. At present, about 75% of the population is in sub-health condition, which seriously affects people’s living standard (Feng et al., 2016). To prevent sub-health and improve immunity has become a hot research topic. Recent studies have found that the gut microbiota can influence host immunity by regulating the level of secondary metabolites and changing intestinal permeability (Dodd et al., 2017). The polysaccharides that cannot be digested and absorbed by the host, it can be digested to short chain fatty acids by the gut microbiota, which promote intestinal absorption and maintain normal intestinal function (Zhou et al., 2020), the body’s anti-inflammatory (Tian et al., 2020) and antioxidant (Liu et al., 2021), and participate in the regulation of the immune system (Zhao et al., 2021).

Polysaccharides from edible fungi have significant pharmacological activity and good biosafety (Yin et al., 2021). Previous scientific studies have found that polysaccharides have a variety of biological activities, such as immunomodulatory, anti-tumor, anti-oxidant. Recent studies have shown that polysaccharide has a good therapeutic effect on the adverse reactions caused by cyclophosphamide, especially for intestinal dysfunction (Li et al., 2021). It has been reported that *Sarcodon imbricatus* polysaccharides protect against cyclophosphamide-induced immunosuppression *via* regulating Nrf2-mediated oxidative stress (Wang et al., 2018b). Zhou et al. (2018) found that tremella polysaccharides showed preventive effect for cyclophosphamide-induced immunosuppressed mice. Protective effects of polysaccharides from *Cordyceps gunnii mycelia* against cyclophosphamide-induced immunosuppression to TLR4/TRAF6/NF-κB signaling in BALB/c mice were observed by Meng et al. (2019). It was found that *Hericium erinaceus* polysaccharide had the protective effect on cyclophosphamide-induced immunosuppression in mice (Wu and Huang, 2021). Therefore, edible fungus polysaccharides could be used as a potential candidate in immune regulation agents.

*Auricularia cornea* var. Li. is genetically stable and high quality of a new variant of *A. cornea* species (Xiao and Kuotan, 2016), which is suitable for large-scale production in China. It is rich in dietary fiber, amino acid, polysaccharides and multi-trace elements, with high nutritional and medicinal value (Wang Y. et al., 2019). Wang et al. (2018a) found that *A. cornea* var. Li. polysaccharides exhibited the hepatoprotective effects against the alcoholic liver diseases through different metabolic pathways. As reported, *A. cornea* var. Li. polysaccharides had good antioxidant capacity (Su and Li, 2020). It has been reported that *A. cornea* var. Li. antidiabetic and antinephritic effects, which is related to the polysaccharide property (Wang D. et al., 2019). *A. cornea* var. Li. has been received more and more attentions owing to its unique phenotype and potential medicinal properties17. However, there is scare papers about the study of *A. cornea* var. Li. polysaccharides regulating immunomodulatory activity.

The objective of this work to analyze the immunomodulatory activity and the mechanism of *A. cornea* var. Li. polysaccharides supplementation in an immunosuppressed mice model induced by cyclophosphamide. The ameliorative effects of *A. cornea* var. Li. polysaccharides on immune system including immune organ indices, immunoglobulin contents, and inflammation cytokines in immunosuppressed mice were studied. In addition, the regulatory effects of *A. cornea* var. Li. polysaccharides on the gut microbiota and their metabolites were analyzed. This study provides useful information for applications of *A. cornea* var. Li. polysaccharides in immune-regulated foods and medicine.

## Materials and methods

### Materials and chemicals

*Auricularia cornea* var. Li. was purchased from the edible fungus base in Heilongjiang province. Mouse tumor necrosis factor-α (TNF-α), interleukin-2 (IL-2), interleukin-4 (IL-4), and interleukin-10 (IL-10) ELISA kit were obtained from Shanghai Jianglai Biotechnology Co., Ltd., (Shanghai, China). Cyclophosphamide was purchased from Shanghai Ryon Biological Technology Co., Ltd., (Shanghai, China). ELISA kits for immunoglobulin (IgA, IgG, IgM, and sIgA) were bought from Nanjing Jiancheng Biological Co., Ltd., (Nanjing, China).

### Isolation and purification of *Auricularia cornea* var. Li. polysaccharides

*Auricularia cornea* var. Li. polysaccharides was extracted and purified following our methods (Kong et al., 2020).

### Animals and experimental design

After 1 week of acclimatization, mice were randomly divided into three groups (12 mice per group), including the control, model and *A. cornea* var. Li. polysaccharides (ACP) groups. The mice in the model and ACP groups were intraperitoneally injected cyclophosphamide with at the dose of 80 mg/kg body weight on Day 7, 8, and 9 to induce immunosuppression (Tang et al., 2018), while the mice in the control group were intraperitoneally injected with the equal volume of 0.9% normal saline. The mice in control and model groups were orally administered 0.9% normal saline, and the mice in ACP group were orally administered 200 mg/kg BW of *A. cornea* var. Li. polysaccharides, once daily for 28 consecutive days (Kong et al., 2020). Afterward, fresh feces from each mouse of the three group were collected immediately, and stored at −80°C. The blood and small intestine were obtained for next experiments. The Ethics Committee of the First Affiliated Hospital of Heilongjiang University of Chinese Medicine approved all animal experiments and protocols (2022060801).

### Determination of the immune organ indices

Thymus and spleen were removed and weighed. The immune organ indices were calculated as the following formula: Immune organ index (mg/g) = immune organ weight (mg)/body weight (g).

### Determination of the immunoglobulin contents in serum

The whole blood was centrifuged at 3,500 rpm at for 10 min to collect serum. The small intestine (60 mg) was weighed, homogenized with sterile saline, centrifuged at 3,500 rpm for 15 min to collect the upper liquid. The contents of immunoglobulin (IgA, IgG, IgM in the serum and small intestine by using according to the manufacturer’s operating instructions (Wuhan Huamei Biotech Co., Ltd.).

### Determination of the cytokine levels in serum

The levels of TNF-α, IL-1β, IL-4, and IL-10 in the mouse serum were determined using assay kits in accordance with the manufacturer’s instructions.

### Gut microbiota analysis of cecal contents

The gut microbiota DNA was extracted and isolated according to the Omega bacterial DNA extraction kit instructions. Specific primers, 338F (5’-barcode + ACTCCTACGGGAGGCAGCA-3’), 806R (5’-GGACTACHVGGGTWTCTAAT-3’), for V3-V4 region of bacterial 16S rDNA were used for PCR amplification. PCR products were quantified on a Microplate Reader (BioTek, FLx800) using the Quant-it PicoGreen dsDNA Assay Kit. Illumina’s TruSeq Nano DNA LT Library Prep Kit was used for library construction. The Agilent High Sensitivity DNA Kit was used to inspect the library on the Agilent Bioanalyzer. Quant-it PicoGreen dsDNA Assay Kit was used to quantify the library on Promega QuantiFluor. Pair-end sequencing of 2 × 250 bp was performed using MiSeq Reagent Kit V3 (600 cycles). After sequencing data was disloaded, low-quality reads were removed, including 3 ‘end adapter, reads with average quality value greater than or equal to 20, and reads containing N bases were removed. Using FastQ-Join software, the off-machine data obtained by pair-end sequencing was spliced into long tags through overlap relationship between reads, The quality control and filtering of the spliced tag are carried out to obtain effective data. Tags identified as chimeras by both Reference and *de novo* were removed using the USearch method (Edgar, 2013), operational taxonomic units (OTUs) were clustered using QIIME software (v1.9.1) (Caporaso et al., 2010). The representative sequence was aligned with the GreenGene database using the UcLUST method (DeSantis et al., 2006; Edgar, 2010). QIIME (V1.9.1) tool was used for alpha diversity analysis. Linear Discriminant Analysis Effect Size (LEfSe) was used to look for statistically difference between groups with biometric identifier (Shi et al., 2021).

### Determination the levels of short-chain fatty acids in cecal content

The content of SCFAs in cecal contents was determined by gas chromatography. The colon contents were accurately weighed into the stool sample box, HALO-F100 stool processor was used to prepare 10% suspension. 500 μL of the suspension was put into a 1.5 mL centrifuge tube, 100 μL of meta crotonic acid was added, and then frozen at −30°C for 24 h. After thawing, proteins and other impurities were removed by centrifugation at 8,000 rpm for 3 min at 4°C; The supernatant was removed and filtered through a 0.22 μm water system filter. Operating conditions are as follows: The initial temperature of the column was 75°C, and the final column temperature was 220°C. The injector temperature was 250°C, the injection volume is 1 μL, the split ratio was 5:1, the carrier gas was high pure nitrogen, and the FID detector was used (Yan et al., 2019).

### Statistical analysis

SPSS 26.0 statistical software was used to analyze all data, and the results were expressed as means ± standard. Using one-way analysis of variance (ANOVA), *P* < 0.05 was considered statistically significant.

## Results

### Effect of *Auricularia cornea* var. Li. polysaccharides supplementation on immune organ indexes in immunosuppressed mice

**FIGURE 1:**
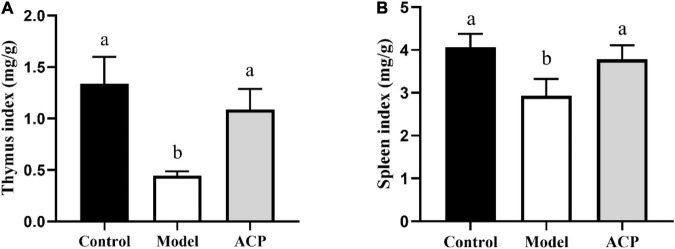
Effect of *Auricularia cornea* var. Li. polysaccharides (ACP) supplementation on immune organ indices in immunosuppressed mice. **(A)** Thymus index and **(B)** spleen index. Values are mean ± SD (*n* = 6 independent experiment). Different superscript letters indicate significant differences (*P* < 0.05) by using one-way analysis of variance, followed by Duncan’s test.

As shown in Figure 1, compared with control group, the cyclophosphamide markedly reduced the thymus and spleen indexes (*P* < 0.05), indicating the immunosuppressed mice model was successfully constructed. After ACP supplementation, the thymus and spleen indexes were significantly increased (*P* < 0.05). These findings implied that ACP supplementation can improve the damage of thymus and spleen caused by cyclophosphamide.

### Effect of *Auricularia cornea* var. Li. polysaccharides on the immunoglobulin contents in immunosuppressed mice

**FIGURE 2:**
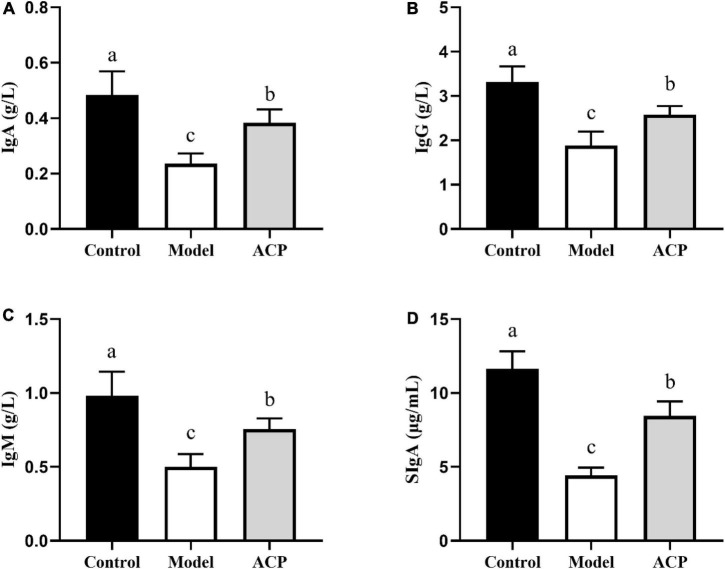
Effect of *Auricularia cornea* var. Li. polysaccharides (ACP) supplementation on immunoglobulin contents in immunosuppressed mice. **(A)** IgA, **(B)** IgM, **(C)** IgG, and **(D)** sIgA. Values are mean ± SD (*n* = 6 independent experiment). Different superscript letters indicate significant differences (*P* < 0.05) by using one-way analysis of variance, followed by Duncan’s test.

The ELISA results of immunoglobulin (IgA, IgG, IgM, and sIgA) contents are represented in Figure 2, the serum contents of IgA, IgG, and IgM in the model group were conspicuously lower than those in the control group. However, compared with model group, the serum contents of IgA, IgG, and IgM was significantly (*P* < 0.05) increased in the ACP group. Furthermore, the sIgA content in the intestinal mucosa of the model group was significantly decreased when compared to the control group, while the sIgA content in the intestinal mucosa of the ACP group was significantly increased when compared to the model group.

### Effect of *Auricularia cornea* var. Li. polysaccharides supplementation on the cytokine levels in immunosuppressed mice

**FIGURE 3:**
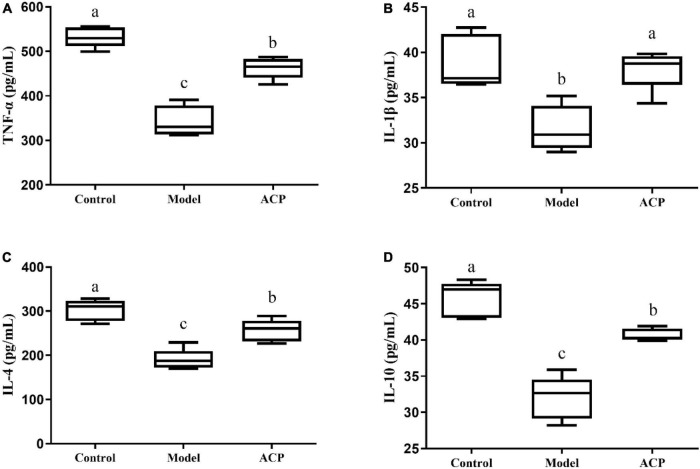
*Auricularia cornea* var. Li. polysaccharides (ACP) supplementation on the levels of serum cytokine in immunosuppressed mice. **(A)** TNF-α, **(B)** IL-1β, **(C)** IL-4, and **(D)** IL-10. Values are mean ± SD (*n* = 6 independent experiment). Different superscript letters indicate significant differences (*P* < 0.05) by using one-way analysis of variance, followed by Duncan’s test.

The levels of TNF-α, IL-1β, IL-4, and IL-10 in serum are reported in this study (Figures 3A–D). A significant decrease (*p* < 0.05) of these five indexes in the model group compared with the control was found. However, there was a significant increase (*p* < 0.05) in the levels of these cytokines in the ACP group compared with the model group. These results indicated that the ACP supplementation could improve the immunosuppressed status by regulating the cytokine levels.

### Effect of *Auricularia cornea* var. Li. polysaccharides supplementation on the α-diversity and β-diversity indexes of gut microbiota in immunosuppressed mice

**FIGURE 4:**
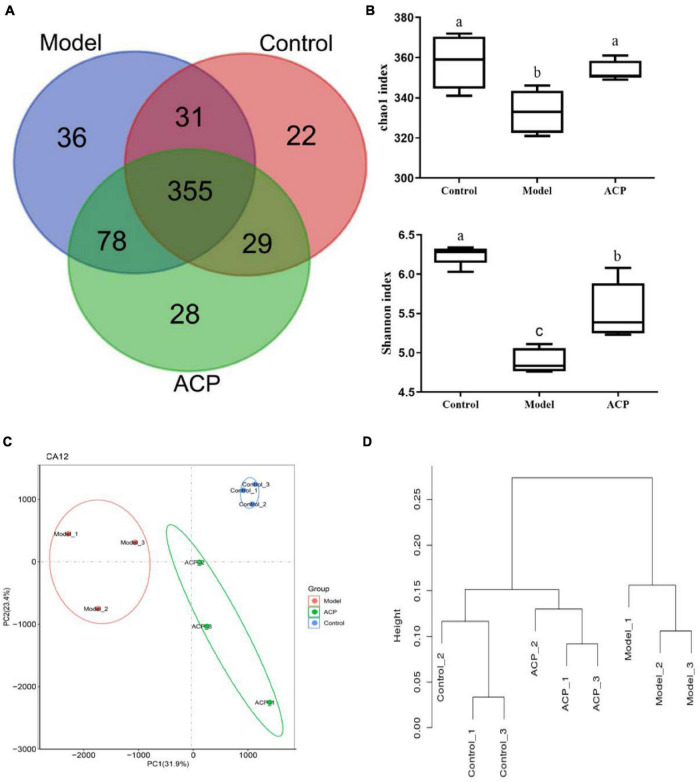
Effect of *Auricularia cornea* var. Li. polysaccharides (ACP) supplementation on the diversity indexes of gut microbiota in immunosuppressed mice. **(A)** Common and unique operational taxonomic units (OTUs) analysis results, **(B)** α-diversity analysis (chao1 index and Shannon index), **(C)** PCA analysis based on OTUs and **(D)** weighted UniFrac distance tree. Values are mean ± SD (*n* = 6 independent experiment). Different superscript letters indicate significant differences (*P* < 0.05) by using one-way analysis of variance, followed by Duncan’s test.

As shown in Figure 4, the number of OTUs shared by the control group, model and ACP groups was 355, and the number of unique OTUs in the control group, model and ACP groups were 22, 36, and 28, respectively. In this study, α-diversity including chao1 index and Shannon index were used to evaluate the richness of each sample. Compared with the control group, chao1 index and Shannon index were significantly decreased in the model group (*P* < 0.05). However, the significant increases (*P* < 0.05) were found in the ACP group when compared to the model group. These findings suggested that ACP supplementation could enhance the richness and diversity of gut microbiota. β-diversity including PCA analysis based on OTUs and system clustering tree of the weighted UniFrac distance, were used to evaluate the differences in microbial community composition among different samples. From the PCoA and UniFrac distance analysis, microbial community composition in the ACP group was closer to the control group than model group. These findings indicated that ACP supplementation shifted the overall structure of the CTX-disrupted gut microbiota toward that of the control group.

### Effect of *Auricularia cornea* var. Li. polysaccharides supplementation on the gut microbiota composition in immunosuppressed mice

**FIGURE 5:**
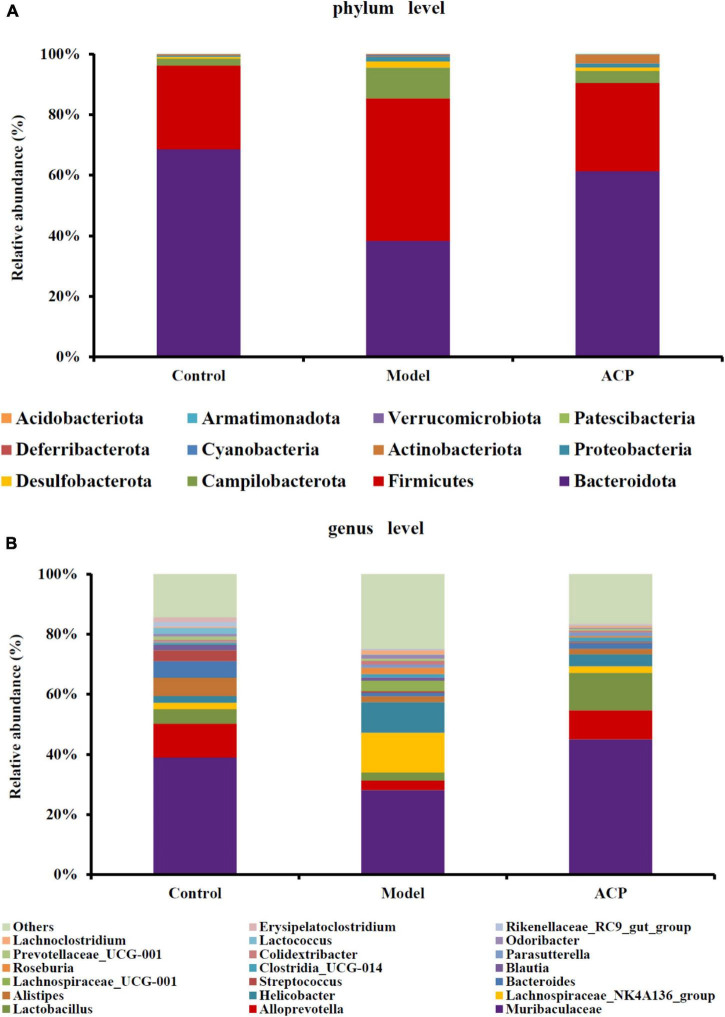
Effect of *Auricularia cornea* var. Li. polysaccharides (ACP) supplementation on the composition of gut microbiota in immunosuppressed mice. **(A)** Phyla level community and **(B)** genus level community.

To further study the gut microbiota composition, the gut microbiota of the control, model and ACP groups was analyzed at the phylum and genus levels. The phylum levels of the gut microbiota are shown as Figure 5A. Bacteroidota and Firmicutes were the dominant taxa. After cyclophosphamide treatment, the relative abundances Bacteroidota and Actinobacteriota were reduced, but the relative abundances of Firmicutes, Desulfobacterota and Proteobacteria were elevated. ACP supplementation restored the alteration of the gut microbiota at the phylum. At the genus level (Figure 5B), a total of 10 genera had an average abundance of more than 1% in the three groups, namely *Muribaculaceae*, *Alloprevotella*, *Lactobacillus*, *Lachnospiraceae_NK4A136_group*, *Helicobacter*, *Alistipes*, *Bacteroides*, *Streptococcus*, *Lachnospiraceae_UCG-001*, and *Blautia*. The relative abundances of *Muribaculaceae*, *Alloprevotella*, *Lactobacillus*, *Bacteroides*, *Streptococcus, Alistipes*, and *Erysipelatoclostridium* in the model group were lower than those in the control group, which were reversed by the ACP supplementation. On the contrary, the relative abundances *Lachnospiraceae_NK4A136_group*, *Helicobacter*, *Lachnoclostridium*, and *Lachnospiraceae_UCG-001* in the model group were increased compared with the control group, which were also shifted to the pattern of the control group after ACP supplementation. These findings indicated that ACP supplementation could effectively regulate the gut microbiota disordered by the cyclophosphamide.

**FIGURE 6:**
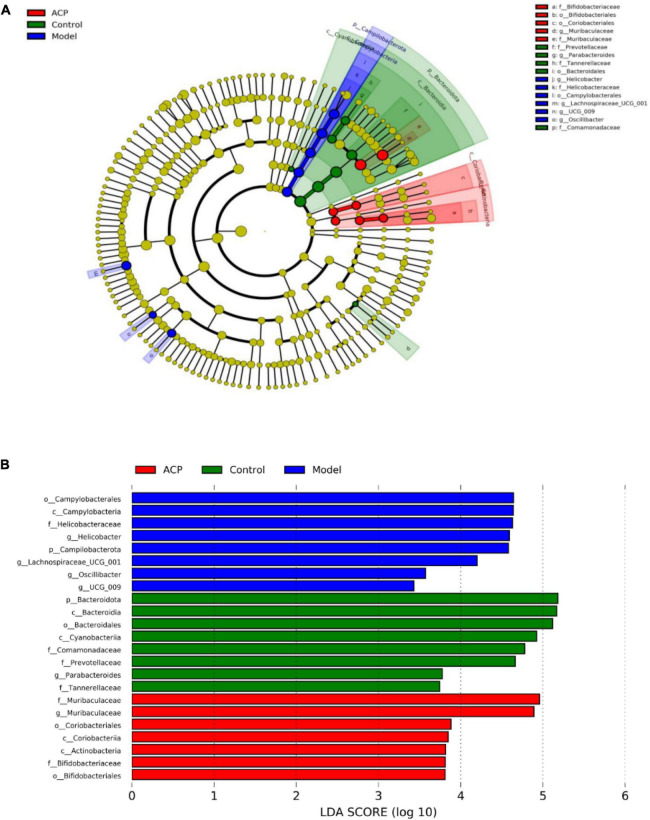
Linear dis-criminant analysis effect size (LEfSe) analysis among the control, model, and *Auricularia cornea* var. Li. polysaccharides (ACP) group. **(A)** Taxonomic cladogram and **(B)** LDA histogram (LDA > 2).

In order to identify the biomarkers among different groups, LEfSe analysis was performed for the three groups, the results are shown in Figures 6A,B. Prevotellaceae, Tannerellaceae, Bacteroidaies, Comamonadaceae and *Parabacteroides* were more abundant in the control group, whereas Campylobacterales, Helicobacteraceae, *Helicobacter*, *Lachnospiraceae_UCG-001*, and *Oscillobacter* were dominant in the model group. Coriobacteriales, Bifidobacteriales, Bifidobacteriaceae, and *Muribaculaceae* were enriched the ACP group.

### Effect of *Auricularia cornea* var. Li. polysaccharides supplementation on the short-chain fatty acids in immunosuppressed mice

**FIGURE 7:**
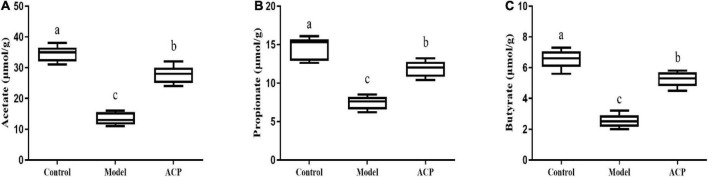
Effect of *Auricularia cornea* var. Li. polysaccharides (ACP) supplementation on the short-chain fatty acids (SCFAs) in cecal contents in immunosuppressed mice. **(A)** Acetate; **(B)** propionate; and **(C)** butyrate. Values are mean ± SD (*n* = 6 independent experiment). Different superscript letters indicate significant differences (*P* < 0.05) by using one-way analysis of variance, followed by Duncan’s test.

The levels of short-chain fatty acids in cecal contents of mice are shown in Figure 7. Our results showed that cyclophosphamide treatment significantly reduced the levels of acetate, propionate and butyrate in cecal contents in mice (*P* < 0.05). The levels of acetate, propionate and butyrate in cecal contents in the ACP group were significantly higher than those of the model group (*P* < 0.05). These results indicated that ACP supplementation could regulate SCFAs contents in intestinal tract.

### Correlation between the main gut microbiota and the immune index

**FIGURE 8:**
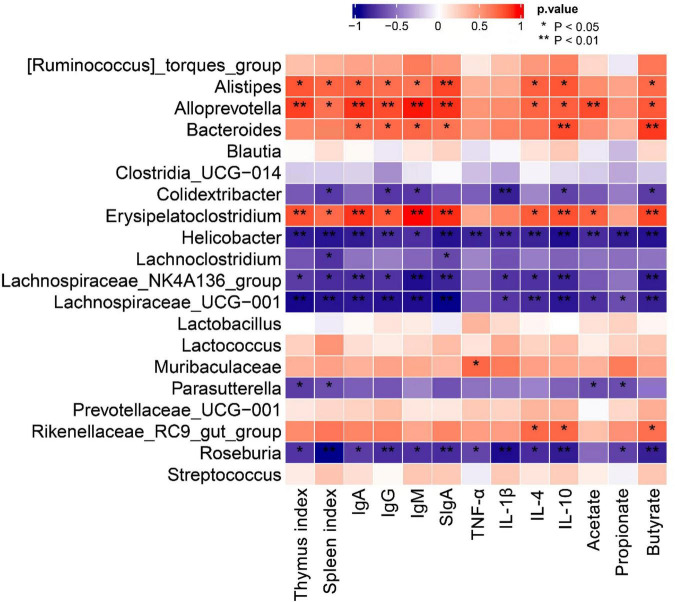
Spearman correlation between genus level communities with the top 20 abundances and immune-related indexes (**P < 0.01, *P < 0.05).

To explore the relationship between gut microbiota and the immune function, Spearman correlation analysis was conducted between and the genus level communities with the top 20 abundances and the immune indexes. As shown in Figure 8, the relative abundance of *Alistipes*, *Alloprevotella*, *Bacteroides*, and *Erysipelatoclostridium* was significantly positively correlated with most of the immune indexes, such as thymus index, spleen index, IgA, IgG, IgM, IL-4, IL-10, and butyrate. The relative abundances of *Lachnospiraceae_NK4A136_group*, *Roseburia, Helicobacter*, and *Lachnospiraceae_UCG-001* showed a significantly negative correlation with most of the immune indexes.

## Discussion

As important immune organs, the thymus and spleen indexes reflect the immune function of the body, which are visual indicators of non-specific immunity (Zhao et al., 2020). In our study, ACP supplementation could increase the thymus and spleen indexes, implying that ACP supplementation is an effective treatment for immunosuppressive status. Cytokines are small molecular peptides and glycoproteins synthesized and secreted by a variety of tissues and cells (mononuclear macrophages, lymphocytes and other immune cells) (Burns et al., 2010). It has many biological functions, such as regulating cell growth, regulating immune response, intercellular signaling and so on. Currently, they can be classified as proinflammatory cytokines, anti-inflammatory cytokines, and cytokines with both proinflammatory and anti-inflammatory activities. Regulating cytokine levels in the body is an effective way to prevent and control inflammatory diseases. T helper cells (TH cells) play an important role in the process of immune response, which are divided into Th1 cells and Th2 cells. Th1 cells mediate cellular immune responses by secreting TNF-α and IL-1β. Th2 cells secrete IL-4 and IL-10 and participate in humoral immunity (Maeda and Shiraishi, 1996; Pellegrini et al., 1996; Tabata et al., 1999; Yoshino et al., 2000). In this study, it was observed that the serum cytokine levels (TNF-α, IL-1β, IL-4, and IL-10) were decreased, indicating that cyclophosphamide could suppress the immune function. However, ACP supplementation could increase the serum cytokine levels. Accumulated evidences have suggested that polysaccharides can improve immune function by modulating cytokine contents. The polysaccharides from *Pueraria lobata* could ameliorate cyclophosphamide-treated mice by elevating the levels of IL-4 and IL-10 (Cai et al., 2022). Sulfated *Cyclocarya paliurus* polysaccharides could improve immune function of immunosuppressed mice by the enhancement of TNF-α and IL-1β productions (Han Y. et al., 2022).

IgG molecules are a cluster of glycoproteins that are important for helping the body fight off invading pathogens (Macpherson et al., 2008). IgM is the first antibody produced in a primary antibody response and is produced primarily by B-1 cells (Kaneko et al., 2006). IgA plays an important role in the protection of immune system due to its obvious distribution between the systemic immune system and mucosal immune system (Mantis et al., 2011). sIgA plays an important role in maintaining a harmonious symbiotic relationship between host and gut microbiota (Peterson et al., 2007). According to the levels of IgA, IgG, and IgM in serum and sIgA in the intestinal mucosa, it can be concluded that ACP could regulate humoral immunity. Zhao et al. (2021) reported that fructo-oligosaccharides improve the immunity of mice with immunosuppression induced by cyclophosphamide through modulating immune globulin.

Intestinal tract is a diversified microbial ecosystem, which is closely related to body health. Studies have found that the composition of gut microbiota is closely related to obesity, diabetes, cardiovascular diseases, inflammatory diseases, body immunity and tumors (Zackular et al., 2013). Therefore, 16S rRNA sequencing was used to compare e the influences of ACP supplementation on gut microbiota of mice with immunosuppression induced by cyclophosphamide. In our study, the relative abundances *Lachnospiraceae_NK4A136_group*, *Helicobacter*, *Lachnoclostridium*, and *Lachnospiraceae_UCG-001* of were increased in the model group. However, ACP supplementation restored the pattern of the gut microbiota. Previous researches have shown that the relative abundances of Lachnospiraceae was positive with the risk of colon cancer (Zeng et al., 2016). Helicobacter is related to chronic gastritis, lymphoma and other diseases (Zhang et al., 2020). These findings was consistent with the results of Bai et al. (2022), who suggested that polysaccharides from Fuzhuan brick tea could elevate immune function by regulating gut microbiota of cyclophosphamide-treated mice. Thus, ACP supplementation could suppress the detrimental gut microbiota to ameliorate the host health. The relative abundances of *Muribaculaceae*, *Alloprevotella*, *Lactobacillus*, *Bacteroides*, *Alistipes* in the model group were lower than those in the control group. However, ACP supplementation reversed this poor situation. *Lactobacillus* is a kind of probiotics, which can enhance immunity, reduce cholesterol, inhibit toxins in the body, antioxidant and anti-tumor (Collier-Hyams et al., 2005; Santos et al., 2018). One of the most frequently detected species in the genus *Alistipes* is *A. onderdonkii*, and it has been suggested that *A. Finegoldii* is a bacteria that alleviates colitis (Parker et al., 2020). *Muribaculaceae*, *Bacteroides*, and *Alloprevotella* are SCFAs-producing bacteria and anti-inflammatory bacteria (Han Y. et al., 2022). *Muribaculaceae* can produce butyrate, which can provide nutrients for intestinal epithelial cells and promote the proliferation of intestinal cells. It has anti-inflammatory, immune-enhancing and anti-tumor effects (Turnbaugh et al., 2006; Walter and Ley, 2011), *Alloprevotella* can produce SCFAs, mainly succinate and acetate, which can improve the intestinal epithelial barrier and prevent inflammation (Wei et al., 2018; Chen et al., 2020). The secretion of SCFAs by *Bacteroides* species can prevent the transport of toxins between the gut lumen and blood, and colon tumor formation in humans (Wang et al., 2022). These results were in accordant with Han Y. et al. (2022), who reported that sulfated *Cyclocarya paliurus* polysaccharides could improve immune function of immunosuppressed mice by modulating gut microbiota.

Short-chain fatty acids (SCFAs) are produced by the fermentation of indigestible carbohydrates, such as resistant starches, by gut microbiota, including acetate, propionate and butyrate (Cook and Sellin, 1998). SCFAs are important fuel for intestinal epithelial cells and can enhance intestinal barrier function (Parada Venegas et al., 2019). Furthermore, the findings suggest that SCFAs, especially butyrate, also play a role in maintaining immune homeostasis (Corrêa-Oliveira et al., 2016). The effects of SCFAs on improving epithelial barrier function and immune function are complex. SCFAs can enhance the differentiation of T lymphocytes into effector T and T-regs lymphocytes by influencing the expression of interferon -γ (IFN-γ), IL-10, and IL-17 (Park et al., 2015; Ratajczak et al., 2019). Our results showed that ACP supplementation reversed the decreases of levels of acetate, propionate and butyrate in cecal contents cyclophosphamide-treated mice. These results were in line with the study of Han C. et al. (2022), who suggested that *Lonicerae flos* polysaccharide had the protective effect of on cyclophosphamide-induced immunosuppression in mice by regulating the SCFAs contents in intestinal tract.

## Conclusion

*Auricularia cornea* var. Li. polysaccharides (ACP) supplementation could improve the spleen and thymus indexes, levels of IgA, IgG and IgM in serum and sIgA in the intestinal mucosa, the levels of TNF-α, IL-1β, IL-4, and IL-10 in the serum. ACP supplementation also could restore gut microbiota to the pattern that is similar with that of the control group with increase of the relative abundances of SCFAs-producing bacteria. Furthermore, the content of SCFAs were increased after ACP supplementation.

## Data availability statement

The datasets presented in this study can be found in online repositories. The names of the repository/repositories and accession number(s) can be found below: https://www.ncbi.nlm.nih.gov/, PRJNA884155.

## Ethics statement

The animal study was reviewed and approved by Ethics Committee of the First Affiliated Hospital of Heilongjiang University of Chinese Medicine (2022060801).

## Author contributions

WS and XK designed the study. MZ performed the experiments. XC and YL wrote the manuscript. YY analyzed the data. All authors contributed to the article and approved the submitted version.
